# Book Review: Cognitive Neuroscience of Natural Language Use

**DOI:** 10.3389/fpsyg.2015.01527

**Published:** 2015-10-06

**Authors:** Giosuè Baggio

**Affiliations:** Language Acquisition and Language Processing Lab, Department of Language and Literature, Norwegian University of Science and TechnologyTrondheim, Norway

**Keywords:** language processing, fMRI, EEG/MEG, ecological validity, methodology, philosophy

Cognitive neuroscientists occasionally grapple with the issue of “ecological validity”: does one's study capture essential aspects of the processes of interest, as they occur in real life? Ecological validity is frequently seen as an impossible standard to meet. In practice, it often receives lower priority than internal consistency, replicability and reproducibility, and control of experimental stimuli, variables and confounds. These desiderata have characterized research in experimental psychology and cognitive neuroscience in the last century. Research on language processing is no exception. The (perceived) gap between naturally occurring behavior and laboratory tasks may be even wider here than in other areas of cognitive science.

The gap may also be wider than necessary, as suggested in *Cognitive Neuroscience of Natural Language Use*, an anthology edited by Roel M. Willems. The book tries to break away from the “impossible standard” view of ecological validity, and restore a more optimistic outlook: there is no hard opposition between carefully controlled experimentation and approximation of real-life processes. One can have one's cake and eat it too, largely thanks to progress in the construction of stimuli and experimental designs, discussed by Willems in the book's Introduction and in the closing section by Hasson and Egidi, and to advances in brain imaging methods, reviewed in a dedicated chapter by Andric and Small. The book does more than reminding its readers that the neuroscientific study of language in (more) ecologically valid conditions is possible in practice. It argues it is theoretically desirable, if our ultimate goal is to understand how the brain engages with discourse, dialogue and even literary texts, not only how it represents and processes words and sentences. These issues are examined in chapters by Kuhlen, Allefeld, Anders and Haynes, and Jacobs.

What makes this collection compelling is its pragmatic stance. Despite its emphasis on “natural” language use, the book's philosophy is quite far removed from the idea that one should observe cognitive processes naturalistically, as they occur in non-laboratory settings (in the tradition of Lewin, Neisser, Bronfenbrenner and others). The latter perspective gives up on the traditional constraints of replicability and control, and is fraught with several methodological difficulties. The book does not adhere to the view that researchers should carry around a controlled task and make it happen in real life (in the tradition of Brunswik), bringing as it were the lab into the real world. This might soon become feasible with the advent of portable neuroimaging technology. The underlying proposal of the book is rather the opposite: bring more real-life features into the laboratory. This is a more modest proposal, but one that is likely to work better than its radical naturalistic predecessors. As several contributions in the book testify, it is already delivering.

The idea of bringing real-world complexity into the lab intercepts the issue of idealization. In much laboratory research on language processing, the stimuli, the task and the experimental setting are *idealized models* of their real-world counterparts. The participant sits in a booth or lays in an MR scanner, secluded from potential conversation partners and from the world of situated action to which language belongs. There she is exposed to isolated “purified” linguistic elements (e.g., syllables or words, often synthesized speech), and must respond to them in ways that bear little resemblance to ordinary verbal behavior (e.g., button presses). The book points to several remedies to this situation: for example, in the domain of language production, the study of spontaneous connected speech in clinical populations, reviewed by Ash and Grossman.

Idealization is all well and good so long as it allows us to understand the laws and principles underlying the phenomena. Eventually this process saturates, and one should seek retreat from idealization. Adding complexity to experimental paradigms allows researchers to assess whether the principles uncovered scale-up or generalize, and to discover new principles. Retreating from the idealizations of traditional psycholinguistics and cognitive neuroscience leads to naturalism. One of the immediate benefits of this move is to create a framework for apparently diverse areas such as M/EEG and fMRI studies of discourse processing, developmental, eye-tracking and ERP research on situated language comprehension, and behavioral and imaging work on coordination and communication games. These fields have grown in recent years, and are well represented in the book, in particular in chapters by Knoeferle, and Stolk, Blokpoel, van Rooij and Toni. The shift also has retrospective value. It gives us a key to interpret a substantial portion of what has been done so far in psycholinguistics and neurolinguistics.

The book thus indicates a clear direction for experimentalists, both historically and conceptually. Should theoreticians and modelers follow suit? Some contributions in the book address modeling issues directly, especially the chapter by Skipper, but there is little discussion of the formal tools that may be instrumental to the success of the enterprise. One may expect a shift from processing models based on formal grammars and lexical semantics to models of discourse, communication and symbolic behavior in general. These models will involve “computational level” analyses (in Marr's sense) based on logics, game theory and related formalisms. Yet formalization issues are hardly foreshadowed in the book. For example, in the chapter on situation models by Kurby and Zacks a connection with formal semantics would be most natural and useful.

The retreat from idealization that inaugurates the naturalistic enterprise poses unique challenges. Integrating theoretical linguistics with cognitive neuroscience has proved exceedingly difficult. Infusing ecologically advanced experimentation with formal models of language use may seem even more daunting. Naturalism needs to make the appropriate idealizations. These will replace the simplifications of traditional laboratory models, but will have to be equally stringent. These will largely determine whether the perspective laid out in the book will be a “softer” version of neurolinguistics, perhaps more oriented toward the humanities and social sciences, or a viable research program along the lines of Marr-style integrative neuroscience.

## Conflict of interest statement

The author of this review and the editor of the collection have previously collaborated for the book chapter Hagoort, P., Baggio, G., and Willems, R. M. “Semantic unification,” in Gazzaniga, M. (Ed.), *The Cognitive Neurosciences* Edition), MIT Press, 2009. The author declares that the research was conducted in the absence of any commercial or financial relationships that could be construed as a potential conflict of interest.

